# Single-cell expression predicts neuron-specific protein homeostasis networks

**DOI:** 10.1098/rsob.230386

**Published:** 2024-01-24

**Authors:** Sebastian Pechmann

**Affiliations:** Sebastian Pechmann Research Lab, Saarbrücken, Germany

**Keywords:** protein homeostasis, neurons, chaperones, Ubiquitin ligases

## Abstract

The protein homeostasis network keeps proteins in their correct shapes and avoids unwanted aggregation. In turn, the accumulation of aberrantly misfolded proteins has been directly associated with the onset of ageing-associated neurodegenerative diseases such as Alzheimer's and Parkinson's. However, a detailed and rational understanding of how protein homeostasis is achieved in health, and how it can be targeted for therapeutic intervention in diseases remains missing. Here, large-scale single-cell expression data from the Allen Brain Map are analysed to investigate the transcription regulation of the core protein homeostasis network across the human brain. Remarkably, distinct expression profiles suggest specialized protein homeostasis networks with systematic adaptations in excitatory neurons, inhibitory neurons and non-neuronal cells. Moreover, several chaperones and Ubiquitin ligases are found transcriptionally coregulated with genes important for synapse formation and maintenance, thus linking protein homeostasis to the regulation of neuronal function. Finally, evolutionary analyses highlight the conservation of an elevated interaction density in the chaperone network, suggesting that one of the most exciting aspects of chaperone action may yet be discovered in their collective action at the systems level. More generally, our work highlights the power of computational analyses for breaking down complexity and gaining complementary insights into fundamental biological problems.

## Graphical abstract

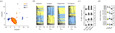

## 1. Introduction

Brain health critically depends on cellular protein homeostasis. In all cell types, a complex regulatory network of protein folding and quality control enzymes [1] such as molecular chaperones [2] and Ubiquitin (Ub) ligases [3] sustains the proteome in a folded and functional state. In turn, failure of protein homeostasis leads to the accumulation of misfolded and aggregation prone proteins [4], and thus ageing-related neurodegeneration [5].

Long studied primarily as a system of protein quality control [6], the protein homeostasis network readily extends in scope to serve as a central hub of regulating cell function [7]. One of the best demonstrations of this is found in neurons where synaptic plasticity can be controlled through the fine-tuning of protein levels between localized protein synthesis [8–10] dependent on cotranslational protein homeostasis [11], and regulated protein turnover [12,13]. Protein homeostasis thus directly couples selective protein quality control with the regulation of neural activity [14]. However, a detailed understanding of protein homeostasis in general, and in neurons in particular, remains elusive.

Importantly, different tissues vary in the chaperones they express [15], as well as differentially predispose to protein homeostasis failure [16]. Chaperones, especially co-chaperones that confer substrate specificity, dramatically increase in numbers with organism complexity [17]. Through tissue-specific expression [15], specialized protein homeostasis networks can both correspond to specific folding needs as well as achieve functional and regulatory fidelity. Yet, a main challenge of understanding protein homeostasis lies in its sheer complexity. This includes a large number of genes involved, a multitude of constraints on the expression of functional proteomes including for stoichiometry [18], function [19] and solubility [20], as well as strong systems-level synergies that achieve pervasive redundancy and resilience in folding [21] and degradation [22] networks. Because protein homeostasis demand and capacity are finely balanced [17], comparative analyses of protein homeostasis in cells of specialized function such as neurons are poised to be particularly informative. To this end, exactly how synapses form and are maintained within neurons also remains incompletely understood [23–25]. From genomic and proteomic studies [26], a set of about 2000 genes likely important for the support of synapses has been curated [27]. Even less is known about the dependencies between synapses and protein homeostasis. For instance, only three E3 ligases could so far be linked to the regulation of pre-synaptic plasticity [28].

Single-cell transcriptomics now afford unprecedented insights into human cell types [29] including in the brain [30,31]. Remarkably, pronounced clusters of individual cells often suggest defined transcriptional cell identities akin to discrete and stable attractor states [32]. Because cell types often differ in the expression of thousands of genes, this has primarily motivated the understanding of cell differentiation [33,34] as a problem of control, i.e. how these cell states are achieved, for instance through inference of the underlying transcription regulatory networks [35]. However, equally critical is to understand how the protein homeostasis network has tightly coevolved with the dynamic proteome demands to render these states functional.

Here, I present a large-scale comparative analysis on data from the Allen Brain Map of the transcription regulation of protein homeostasis at single-cell resolution across the human brain. This effort is entirely complementary to related experimental work. Protein homeostasis networks of distinct and characteristic composition were found for excitatory neurons, inhibitory neurons and non-neuronal cells. Moreover, specific sets of chaperones and Ub ligases in these specialized networks were identified to be directly transcriptionally coregulated with the synaptome, thus suggesting possible functional links and candidate genes for future experiments. Finally, the high interconnectivity of the chaperone network more than individual interactions were found evolutionarily conserved, underlining the importance of understanding protein homeostasis at a systems level.

## 2. Methods

### 2.1. Data and code availability

Project data and computer code generated for this project are available at https://www.github.com/pechmannlab/neuroPN and archived on Zenodo at https://doi.org/10.5281/zenodo.10171304.

### 2.2. Data sources

Single-cell mRNA expression data were obtained from the Allen Brain Map (https://portal.brain-map.org/). Specifically, the processed and clustered human brain tissue single-nucleus (snRNA-seq) expression data from multiple cortical areas [30] were analysed together with a comparative analysis of single-cell expression data of the human, mouse and macaque dorsal lateral geniculate nucleus, the brain region that relays visual information to the primary visual cortex [36]. Only cells with cluster assignments were analysed, resulting in a set of 48 642 cells in 131 clusters. Gene annotations were obtained from the literature. An encompassing set of around 2000 synaptome genes as an initial list of candidate genes linked to synapse formation, maintenance and plasticity was obtained from the SynaptomeDB database [27]. A list of human genes encoding subunits of ion channels was taken from the ModelDB database [37]. A curated list of human chaperones [15,38] was obtained from the Proteostasis Consortium [39], and a list of genes in the human Ubiquitin-dependent degradation system was compiled from the hUbiquitome [40] and UbiNet2.0 [41] databases.

### 2.3. Data analysis

All analyses were limited to genes whose annotations uniquely mapped to corresponding protein sequences in the non-redundant Uniprot reference proteomes [42]. Expression levels were normalized to counts per million. Differentially expressed and variable genes were detected as implemented in Seurat [43]. Genes with a greater than twofold change relative to their overall median expression were highlighted as very differentially expressed. Dimensionality reduction of cell expression profiles was performed with UMAP [44]. Percentages of differentially expressed genes were used to show how much of transcriptomes was substantially remodelled between cell classes. Cumulative expression was used to approximate the overall activity of cellular networks such as a total folding or degradation capacity.

### 2.4. Coexpression networks

Coexpression networks were generated from interacting gene pairs defined based on highly correlating expression profiles. All interactions considered in this work were therefore coexpression interactions. To circumvent the problem of missing values in single-cell expression data, the data were transformed to a vector of pairwise comparisons that by themselves were more robust [45]. Specifically, the per gene expression profile across *n* cell clusters was replaced by a vector of *n* × (*n −* 1)/2 fold changes from pairwise differential expression tests. Pearson correlation coefficients between gene pairs were then used to construct coexpression networks [46]. Thresholds to call coexpression interactions were arbitrary and reflected a trade-off between stringency and sufficient data points for further analysis; in the full data the top 5%, and in the smaller species-specific coexpression networks the top 20% of correlating gene pairs were called as interacting. Importantly, the top interaction scores derived from a long upper tail of a non-uniform distribution of all pairwise correlations thus were strongly non-random. Furthermore, to construct selective coexpression networks, only genes that were systematically overexpressed by at least 20% in the target cells versus a control, for instance in all neurons versus non-neuronal cells, were considered. Last, to increase confidence and limit networks to genes that may partake in feedback interactions, all motifs of size *n* = 3 within coexpression networks were enumerated as previously described [47]. Only genes contained in the motifs, i.e. with multiple systematic interactions, were subsequently used to construct final networks. Network modules that identify highly interconnected subnetworks were detected with the Python Networkx library.

### 2.5. Network evolution

Orthologue assignments for the reference proteomes without isoforms [42] between human, macaque and mouse were recomputed with OMA [48]. Comparative expression data for these three species were available from [36]. Coexpression networks were generated as described above, and corresponding interactions between orthologous gene pairs that were present in all three species-specific networks were considered as conserved. To understand any selective forces on the conservation of coexpression interactions, null-models were generated through 1000 degree-preserving randomizations of the species networks with the R package BiRewire [49]. Finally, a conservation score was defined as the per cent difference between the numbers *N* of true and randomly conserved interactions as *conservation* = ((*N*_true_/*N*_random_) − 1) × 100. Relative connectivity was defined as the observed network degree relative to the corresponding averages resulting from randomized networks of the same sizes.

## 3. Results

**Figure 1.**
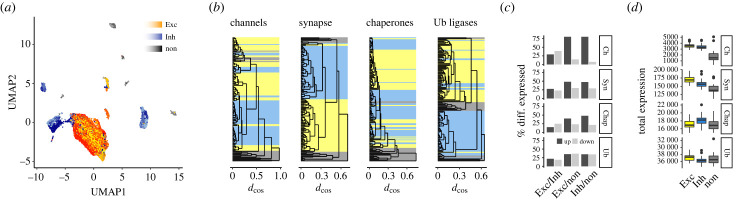
Expression of neuron-specific protein homeostasis networks in the brain. (*a*) UMAP plot of the single-nucleus mRNA expression profiles of 48 642 cells in the human brain from the Allen Brain Map. Cells separated well into the cell classes of excitatory neurons (Exc), inhibitory neurons (Inh) and non-neuronal glia cells (non). (*b*) Comparison of the expression of protein homeostasis and synaptome genes. Dendrograms built from the hierarchical clustering based on cosine distance *d*_cos_ show similarities in the expression profiles of ion channels (*n* = 42), synaptome genes (*n* = 1746), chaperones (*n* = 215) and Ubiquitin ligases (*n* = 376). The corresponding cell classes are highlighted for excitatory neurons (yellow), inhibitory neurons (blue) and glia (grey). (*c*) Differential expression of protein homeostasis and synaptome genes between excitatory neurons, inhibitory neurons and non-neuronal cells. For each pairwise comparison are indicated the percentages of genes significantly up- and downregulated. Pairwise comparisons were performed between all excitatory and inhibitory neurons, between excitatory neurons and non-neuronal cells, as well as between inhibitory neurons and non-neuronal cells. (*d*) Total expression of protein homeostasis and synaptome genes in excitatory neurons, inhibitory neurons and non-neuronal cells.

The large-scale mapping of mRNA expression at single-cell resolution has opened unprecedented opportunity to investigate shared and specialized characteristics of cell types. Here, single-cell expression data from the Allen Brain Map collected across multiple brain regions were analysed to better understand whether neurons express specialized protein homeostasis networks that associate with synapse formation and maintenance. Specifically, by combining two recent studies on the systematic mapping of multiple cortical areas [30] and the dorsal lateral geniculate nucleus [36], expression profiles of 48 642 neuronal and non-neuronal human cells partitioning into 131 clusters were obtained. Visualization in reduced dimensions supported the validity of merging these two studies, and highlighted a good separation between excitatory neurons, inhibitory neurons and non-neuronal glia cells as overarching cell classes, thus supporting the overall high quality of the data (figure 1*a*).

As expected, the expression profiles of ion channels as a main characteristic of neurons strongly differentiated between excitatory neurons, inhibitory neurons and non-neuronal glia cells (figure 1*b*). The synaptome as the set of genes involved in synapse formation and maintenance [27] is less defined, yet also readily partitioned cells by their class (figure 1*b*). Within cell classes, for instance in comparison between excitatory neurons, differences were smaller as evident from shorter branch lengths in the cluster dendrograms (figure 1*b*). After thus confirming that the differences in expression (figure 1*a*) indeed traced back to genes especially important for neuronal identity, I asked whether such systematic differences also extended to the corresponding protein homeostasis networks. To test this, I compared the expression profiles of the core protein homeostasis network, namely the genes encoding the chaperone-mediated protein folding, and the Ubiquitin-dependent protein degradation systems (see Methods). Strikingly, the expression profiles of both chaperones and Ub enzymes were almost equally strongly predictive of the cell class, with a little more variability for the chaperones and an even stronger signal for the Ub enzymes (figure 1*b*). Of note, other, unrelated genes could similarly classify brain cells, suggesting very strongly defined cell identities far beyond the expression of known marker genes.

In direct comparison, ion channels were, naturally, strongly overexpressed in neurons (figure 1*c*,*d*). Percentages of differentially expressed genes highlighted the fraction of genes that were substantially remodelled (figure 1*c*), while the total expression approximated overall activity in characteristic transcriptomes (figure 1*d*). The synaptome already painted a more varied picture. While many of its genes were overrepresented in neurons, a sizeable fraction was instead found enriched in glia cells (figure 1*c*), even if the overall synapotome expression in glia was clearly lower (figure 1*d*). The definition of the synaptome in its current form may harbour potential for further context-dependent refinement. Interestingly, a large fraction of chaperones was also overexpressed in neurons, hinting at a prominent role of chaperone-mediated protein homeostasis in neurons (figure 1*c*). By contrast, some Ub enzymes were enriched in neurons and others in glia cells (figure 1*c*). Moreover, several chaperone and Ub ligase genes were differentially expressed between excitatory and inhibitory neurons. Taken together, these observations established that excitatory and inhibitory neurons systematically express characteristic protein homeostasis networks of distinct composition.

**Figure 2.**
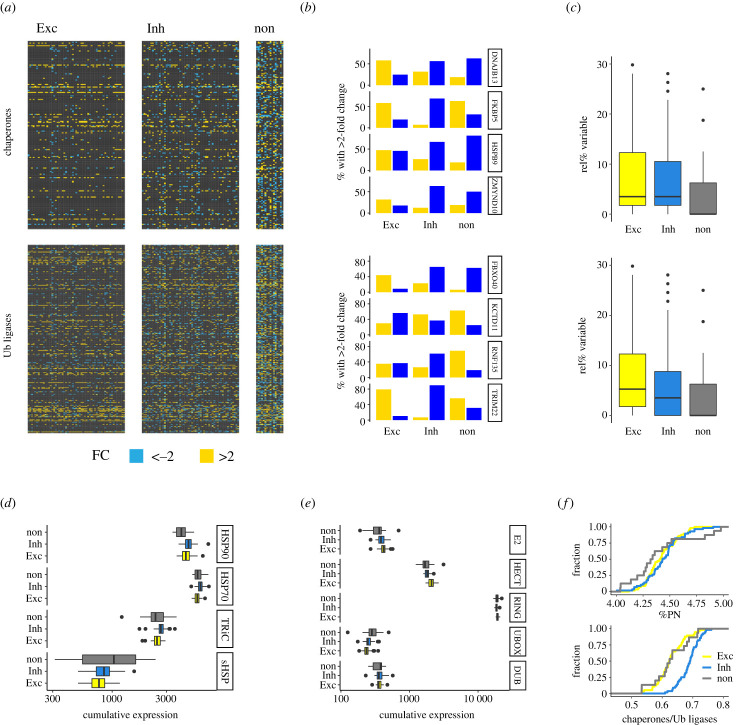
Variability and systematic adaptations in the expression of neuronal protein homeostasis. (*a*) Expression heatmaps of chaperone and Ub ligase mRNA levels relative to their per gene median across all cells. Fold changes (FC) of up- or downregulation relative to overall median expression greater than two are shown in yellow and blue, respectively. Columns denote individual cell clusters, and reported are average values for each cluster. Within the classes of excitatory neurons, inhibitory neurons and non-neuronal glia, columns representing cell types are ordered following the hierarchical clustering based on their full genome expression similarity. (*b*) Percentages of exemplary chaperone and Ub ligase genes that were at least twofold over- or underexpressed relative to their median expression. Corresponding to the heatmaps, substantial variability was observed for a large number of protein homeostasis genes even within classes of related cell types. (*c*) Distributions of the percentages of gene variability. For each chaperone or Ub ligase the percentage of cell clusters is shown within which the gene was identified as variably expressed. Chaperone genes were found particularly variable in excitatory neurons compared to their expression in the other cell classes. (*d*) Cumulative expression by enzyme family. To test if some variability of individual protein homeostasis genes is buffered at the systems level, cumulative expression levels for groups of genes that encode structurally, and thus often functionally, related proteins were computed. Shifts in the distributions of cumulative expression levels suggested systematic adaptation in protein homeostasis network composition. (*e*) Distribution of the total expression of core protein homeostasis network (PN) gene expression relative to the full genome for all cell clusters and grouped by cell class. (*f*) Distribution of the ratio of chaperone to Ub ligase expression for all cell clusters and grouped by cell class. Inhibitory neurons expressed a higher relative content of chaperones.

How representative are these overall trends for individual cell clusters? Visualization of twofold deviation from median expression highlighted extensive variability between clusters that was otherwise masked by summary statistics (figure 2*a*). As reported previously [15], some chaperones were constituently expressed yet many others exhibited variable cell type specific expression. The same could be observed for Ub ligases (figure 2*a*). Importantly, next to the systematic differential expression of protein homeostasis genes between cell classes, substantial variability was equally detected between different excitatory and inhibitory neurons, respectively (figure 2*a*).

Several noteworthy examples stood out (figure 2*b*). The HSP90 co-chaperone *ZMYND10* was preferentially found overexpressed in excitatory neurons and less frequently in inhibitory neurons and glia. By contrast, the HSP90 co-chaperone *FKBP5* was overexpressed in most excitatory neurons and glia, but not often in inhibitory neurons. The ER-localized HSP40 *DNAJB13* was found overexpressed only in excitatory neurons, while the sHSP *HSPB9* was overexpressed in half of the excitatory neurons, and depleted from the other half (figure 2*b*). Similarly, the E3 Ub ligase *FBXO40* was overexpressed in excitatory neurons, whereas *KCTD11* was depleted from most excitatory neurons but overexpressed in inhibitory neurons and glia. Another E3 ligase, *FNF135*, was found to vary substantially between excitatory neurons while depleted from inhibitory neurons, and *TRIM22* was mostly depleted only in inhibitory neurons (figure 2*b*). Remarkably, the same protein homeostasis genes were expressed more variably in neurons, especially excitatory neurons, than in non-neuronal cells (Wilcoxon–Mann–Whitney test; Exc versus non: *p*_chap_ = 0.000049 and *p*_ub_ = 0.000185; Inh versus non: *p*_chap_ = 0.000038 and *p*_ub_ = 0.0411) (figure 2*c*). These data suggest that individual cell type specific expression and pervasive variability are fundamental characteristics of protein homeostasis.

The presence or the absence of individual protein homeostasis enzymes can be compensated by the expression of functionally redundant genes. To test whether the variable expression of some chaperones and Ub ligases was buffered at the systems level, I next looked at their cumulative expression. Chaperone families such as HSP90 or HSP70 type chaperones perform distinct tasks in the protein folding cycle [2,17], and thus comprise reasonable groupings. Importantly, the cumulative expression of chaperone families highlighted several interesting systematic adaptations. Specifically, the post-translationally acting stress chaperones from the HSP90 family were on average expressed more, from the sHSP family less in neurons compared to non-neuronal cells (figure 2*d*). By contrast, the protein biogenesis HSP70s and the TRiC/CCT chaperonin subunits were systematically enriched in inhibitory neurons only (figure 2*d*). Coordinating E2 ligases and deubiquitination enzymes (DUBs) perform unique tasks in the Ubiquitin-dependent degradation cycle, but groups of structurally related E3 ligases differ predominantly in their activation mechanisms rather than cellular function. Interestingly, E2 ligases were systematically more expressed in neurons compared to glia, UBOX-family ligases depleted in neurons and HECT ligases enriched only in excitatory neurons (figure 2*e*). The vast majority of E3s belongs to the family of RING ligases for which no pronounced difference was observed (figure 2*e*).

Finally, the total expression of core protein homeostasis genes was found at a stable 4–5% of total genome expression irrespective of cell type or class (figure 2*f*). However, the relative ratio between chaperone and Ub enzyme expression revealed a strong and systematic shift of a much higher relative chaperone content in inhibitory neurons (figure 2*f*). This observation traced back to the higher chaperone expression (figure 2*d*) and lower Ub enzyme expression (figure 2*e*) in inhibitory neurons. Thus, next to pronounced variability and cell-specific expression, these results revealed systematic adaptations in composition of the protein homeostasis networks in different cell classes.

**Figure 3.**
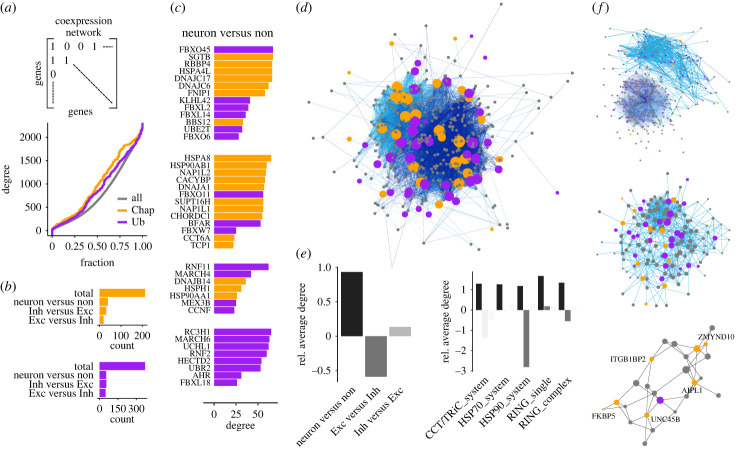
Coregulation of protein homeostasis and the synaptome. (*a*) Coexpression networks were built from coregulated gene pairs with highly correlated expression profiles. All interactions considered are thus coexpression interactions between genes. In the resulting networks, chaperones and Ubiquitin enzymes were characterized by above average degree distributions. (*b*) Selective coexpression networks were generated for all neurons, excitatory neurons and inhibitory neurons. These were classical coexpression networks with the additional requirement that all nodes were overexpressed relative to a background, for example, genes overexpressed in excitatory neurons compared to inhibitory neurons. The numbers of chaperones and Ubiquitin enzymes interacting with the synaptome in each network, which is labelled by both selected subset and background, are shown. (*c*) Protein homeostasis genes enriched in neurons and coregulated with the synaptome are shown together with their network degree and grouped by network modules. Network modules highlight especially interconnected subnetworks that often reveal functionally coupled genes. (*d*) Network representation of the coexpression network in neurons. The node size of protein homeostasis genes scales with their degree and highlights the presence of both highly connected and central to the network as well as less connected and peripheral chaperones and Ub ligases. (*e*) Relative network degree of protein homeostasis genes normalized by a randomization control and shown by cell class as well as corresponding enzyme family. The protein homeostasis–synpase network is, as expected, much denser in neurons than non-neuronal cells, but in direct comparison less dense in excitatory neurons compared to inhibitory neurons. (*f*) Selective coexpression network of excitatory neurons. Shown are the full network coloured by modules (top), the module containing the most HSP90 co-chaperones (middle) and the subgraph containing only nodes connected to HSP90 co-chaperones (bottom).

Having established that neurons express protein homeostasis networks of distinct compositions, I next sought to test if these were linked to synapses as their main function. Coexpression networks are a powerful tool for the discovery of coregulated genes that may be functionally coupled [46]. Here, selective coexpression networks were generated with the additional condition that only genes systematically overexpressed in target cells versus a control, for instance in neurons versus glia, were considered (see Methods). Of note, all subsequently reported interactions are coexpression interactions between genes, not directly detected protein–protein interactions. Owing to their central roles in cellular homeostasis, both chaperones and Ub enzymes had on average a higher degree of coexpression interactions than the rest of the genome (figure 3*a*). Importantly, about 10% of the chaperones and Ubiquitin enzymes were both enriched in neurons and coregulated with the synaptome (figure 3*b*). Thus, only a specific and select subset of protein homeostasis genes directly associated at the level of transcription with genes involved in synapse plasticity.

The selective coexpression network of neurons compared to non-neuronal cells organized into several network modules, i.e. highly interconnected subnetworks, that highlighted chaperones and Ub ligases coupled to the synaptome (figure 3*c*). The grouping revealed by these modules suggested interesting combinations of chaperones and Ub enzymes that may act in concert. Strikingly, some of the thus identified genes were previously known to be directly involved in synapses, such as the E3 ligase *FBXO45* central to neuromuscular synaptogenesis. Others closely clustered, such as the HSP40s *DNAJC6* and *DNAJC17*, the HSP70 *HSPA4L* and the HSP90 co-chaperone *SGTB*, all involved in conferring substrate specificity (figure 3*c*). Another module contained three histone chaperones *NAP1L2*, *SUPT16H* and *NAP1L1* as well as two subunits of the TRiC/CCT chaperonin together with *HSP8A*, the main link to chaperone-mediated autophagy. While neither of these chaperones is neuron-specific, their context-specific coupling of chromatin regulation, cotranslational protein folding and autophagy as alternative to Ubiquitin-dependent degradation may be.

Overall, the resulting coexpression network between core protein homeostasis genes and the synaptome was dense and highly interconnected (figure 3*d*). The relative connectivity of protein homeostasis genes, here defined as the number of coexpression interactions normalized by a randomized control (see Methods), was higher than expected in neurons (figure 3*e*). This observation hinted at an elevated role of redundant and resilient protein homeostasis in neurons compared to non-neuronal cells. Furthermore, the direct comparison of excitatory and inhibitory neurons specified a slightly higher relative connectivity in inhibitory neurons and a clear reduction in the relative connectivity of protein homeostasis genes in excitatory neurons (figure 3*e*). Breaking down these trends by enzyme family, the higher relative connectivity in neurons compared with glia was general. However, the reduction in relative connectivity observed in excitatory neurons traced down to the HSP90 system (figure 2*e*). Moreover, by zooming in on the selective coexpression network in excitatory neurons relative to inhibitory neurons, a network module containing several HSP90 co-chaperones could be identified (figure 2*f*), thus suggesting indeed a functional link between them.

In conclusion, select subgroups of protein homeostasis genes were found not only systematically overexpressed in neurons but also transcriptionally coregulated with genes important for synapse formation and maintenance. Some of these genes were already known to be important for synapses, but the role of most has not been characterized in neurons. In addition, several systematic trends could be observed. HSP90 co-chaperones characterized by lower connectivity in excitatory neurons were consistently rediscovered in the same network module, likely because of intertwined functions. Naturally, the resolution of single-cell mRNA expression data is not sufficient to posit on mechanisms of protein interactions. However, the protein homeostasis genes identified should be some of the strongest candidates for future experiments into neuronal protein homeostasis given the current data.

**Figure 4.**
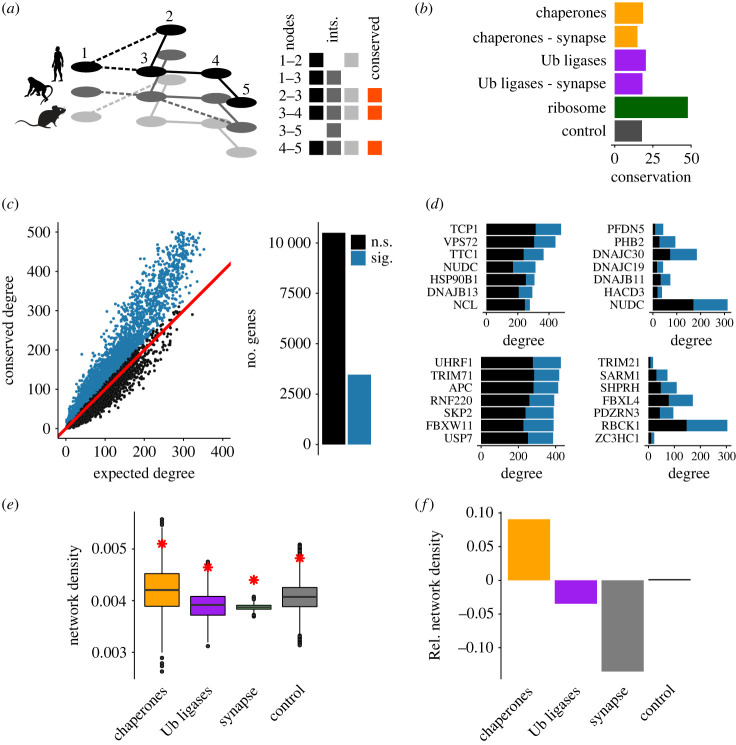
Evolutionary conservation of protein homeostasis coexpression networks in neurons. (*a*) Species-specific coexpression networks from human, macaque, and mouse single-cell expression data were used to test for evolutionary conservation. The presence or the absence of coexpression interactions (ints.) between orthologous gene pairs (nodes) is evaluated. If a corresponding interaction is present in all three species-specific coexpression networks, then the interaction is considered conserved. Conserved interactions are marked with solid lines and non-conserved interactions with dashed lines. In the summary schematic, interactions present in all three networks are additionally highlighted in red. (*b*) Interactions between ribosomal genes as intrinsic control were well conserved, thus suggesting strong validity of this evolutionary analysis. Chaperone and Ubiquitin enzyme interactions were more conserved than chance, but not more conserved than a background signal of interactions between random genes. Naturally, even ‘random' genes carry important functions in the cell that are conserved between closely related species. The conservation score itself is the per cent difference of conserved interactions in the true versus randomized networks (see Methods). (*c*) Observed versus expected conservation of coexpression interactions. The interactions of only about a quarter of all genes were significantly conserved. (*d*) Protein homeostasis genes with the most conserved interactions (left) and the highest ratio of conserved to expected interactions (right). (*e*) Observed network density of conserved interactions (red star) relative to the distributions of randomized controls for chaperone, Ubiquitin enzyme, synaptome and control networks. (*f*) Relative network density obtained through normalization by a randomized control of the same network sizes afforded the direct comparison and highlighted a conserved higher network density in the chaperone network.

Last, because evolutionary conservation is one of the strongest indications of functional importance, I sought to explore whether the functional enrichment and coregulation of protein homeostasis genes were evolutionarily conserved. To test this, species-specific coexpression networks were constructed for a smaller dataset of the dorsal lateral geniculate nucleus [36] that contained expression data for human, macaque and mouse (figure 4*a*) (see Methods). As a control, I first looked at ribosomal genes that are known to be functionally coupled and conserved. Importantly, coexpression interactions between ribosomal genes were indeed strongly evolutionarily conserved compared to a randomized control (figure 4*b*), suggesting informative power of such an analysis with the available data. However, interactions of chaperones and Ub enzymes were not more conserved than those of a random set of control genes (figure 4*b*). This was not surprising given the promiscuous role of chaperones and Ub ligases that often interact with hundreds of client proteins, as well as a baseline signal reflecting the overall close relationship between the species.

About a quarter of genes had more conserved coexpression interactions than expected by chance (figure 4*c*). Of those, the chaperones with the highest number of conserved interactions included some of the most central chaperones such as the HSP90 *HSP90B1* and the TRiC/CCT subunit *TCP1* (figure 4*d*). By contrast, the chaperones with the highest ratio of conserved to expected interactions were the Prefoldin subunit *PFDN5* as well as the three HSP40s *DNAJC30*, *DNAJC19* and *DNAJB11* that are part of the triage of cotranslational protein folding. Similarly, the Ub ligases with the highest conserved degree included two E3s initially not linked to synapse biology, namely UHRF1 important for chromatin regulation and *TRIM71* important for miRNAs, but also *APC* and *RNF220* that play important roles in Wnt-signalling, implicated in neurogensis. Of the Ub enzymes with the highest ratio of conserved to expected interactions, only *SARM1* is known to be important for protein degradation in axons while the others have not yet been implicated in neuron-specific processes (figure 4*d*).

Finally, protein homeostasis is characterized by strongly interconnected and redundant networks. The network density, here defined as the fraction of conserved coexpression interactions within a gene set, was found systematically higher than chance for all networks of chaperones, Ub enzymes, the synaptome and a randomized control (figure 4*e*). Normalizing the observed network densities by the randomized controls to directly compare these networks of different sizes revealed a higher than expected relative network density of the chaperone network compared to the Ub network, and much higher than the network of the synaptome genes (figure 4*f*). Interestingly, while both chaperones and Ub enzymes displayed on average a higher degree (figure 3*a*), the conserved density was only higher in the chaperone network. It is well known that the chaperone network is especially modular, redundant and dynamic, even more so than the Ub system that is more linear and hierarchic. The higher evolutionarily conserved relative network density across human, macaque and mouse transcriptomes highlighted the importance of systems level effects in addition to individual interactions especially for the chaperone system.

## 4. Discussion

This computational analysis of single-cell mRNA expression across the human brain has revealed protein homeostasis networks of distinct composition in excitatory neurons, inhibitory neurons and non-neuronal cells. Remarkably, several protein homeostasis genes directly associated, through transcriptional coregulation, with genes involved in synapse formation and maintenance. While the thus identified chaperones and Ub ligases promise to be excellent candidate genes for future perturbation and characterization, the complexity and challenges of understanding protein homeostasis were underlined by beyond average variability in chaperone and Ub ligase expression as well as selection on systems level characteristics.

A major current limitation lies in mRNA expression levels as proxy for the inference of protein homeostasis networks, given that mammalian cells use extensive translational regulation [50,51]. Nonetheless, especially systematic and pronounced changes at the transcript level are strongly indicative of corresponding protein levels. The continued development of modern methods [52], especially for the quantification of protein abundances at single-cell resolution, will without doubt further revolutionize insights and progress. Until then, there is tremendous merit in analysing already available data within their limits.

One such limitation is an insufficient resolution to imply physical protein interactions between chaperones or Ub ligases and their clients from the transcriptional coregulation of their genes. To rationalize any regulatory interdependencies as well as how redundancies give rise to extraordinary resilience in protein homeostasis, interaction specificities in the network have to be better understood. Fortunately, many aspects of protein behaviour can be increasingly inferred from their sequences and structures, such as their propensity for stress-granule formation [53] or interaction with chaperones [54]. Moreover, not all chaperone or Ub ligase interactions will involve functional regulation. Some of the most folding-challenged proteins such as acting and tubulin simply depend on specific chaperones for their folding needs [55,56]. In other cases, titration of shared chaperone or degradation resources that prioritize the folding and quality control of some but not of other proteins may be much more exploited for systems level regulation than currently appreciated.

Despite tremendous achievements so far, further progress is also needed towards a rational understanding of synapse formation and maintenance [25]. Because neurons still cannot be easily cultured in the laboratory and thus subjected to all the technical advances of modern genomics, perturbation experiments are often cumbersome and limited to whole organism viable variants. Even more important become the use of already available technology and data together with integrated computational analyses that can help break down complexity and generate novel hypotheses. For instance, the observed fundamental shift in the division of labour between chaperone-mediated protein folding and Ubiquitin-dependent protein degradation in excitatory and inhibitory neurons (figure 2*f*) is remarkable because already an imbalance of excitatory and inhibitory neurons has contributed to ageing [57] and neurodegeneration [58]. Next to understanding any imbalances in disease conditions, it is intriguing to speculate on the possibility of autophagy as an alternative degradation mechanism to balance the fluxes of protein folding and degradation in different types of neurons.

Finally, the above average variability of chaperone and Ub ligase gene expression is pronounced and noteworthy. Gene expression in general is inherently stochastic [59], as now quantifiable by single-cell genomics [60]. Moreover, some of that variability may be further amplified through translational heterogeneity [61]. The suppression of expression noise is costly [62], and some fluctuations are even functional, for instance in cell-fate decisions [63] or during differentiation [64]. However, a robust cell identity has to persevere in light of perturbations, for instance though robust control of transcription regulation [65]. Equally importantly, the expression of a functional proteome will have to be robustly supported by sufficient protein homeostasis capacity [17], even in light of fluctuations. This is often a limitation in disease linked to protein misfolding [4], but can also be a challenge in genome engineering or cellular reprogramming efforts, especially towards supporting synthetic non-native states [32]. The presented insight of cell-specific protein homeostasis networks prepares for future mechanistic whole-cell models of protein homeostasis to address these challenges and better understand the role of protein homeostasis in health, ageing and neurodegeneration.

## Data Availability

Data and computer code can be freely accessed at https://www.github.com/pechmannlab/neuroPN. All data and computer code can be accessed at www.github.com/pechmannlab/neuroPN and on Zenodo at https://doi.org/10.5281/zenodo.10171304 [66].
